# Epigenetic Age Feedback as a Catalyst for Sustained Lifestyle Change: One-Year Results from the EU iHelp Study

**DOI:** 10.3390/epigenomes10020022

**Published:** 2026-04-01

**Authors:** Nien-yu Yang, Yicong Huang, Chaewon Park, Te-Min Ke, Graham Tilston, George Manias, Dimosthenis Kyriazis, Jon Young, Susan Hart, Graham Fulford, Artitaya Lophatananon, Kenneth R. Muir

**Affiliations:** 1 Division of Population Health, Health Services Research and Primary Care, School of Health Sciences, Faculty of Biology, Medicine and Health, The University of Manchester, Manchester M13 9PT, UK; sunny.yang@manchester.ac.uk (N.-y.Y.); yicong.huang@manchester.ac.uk (Y.H.); chaewon.park@manchester.ac.uk (C.P.); graham.tilston@manchester.ac.uk (G.T.); artitaya.lophatananon@manchester.ac.uk (A.L.); 2 Department of Radiation Oncology, Chi Mei Medical Center, No. 901, Zhonghua Road, Yongkang District, Tainan City 710, Taiwan; b30932@mail.chimei.org.tw; 3 Department of Digital Systems, University of Piraeus, 18534 Piraeus, Greece; gmanias@unipi.gr (G.M.); dimos@unipi.gr (D.K.); 4 The Graham Fulford Charitable Trust, 66b Smith Street, Warwick CV34 4HU, UK; jon@psatests.org.uk (J.Y.); susan@psatests.org.uk (S.H.); grahamfulford47@gmail.com (G.F.)

**Keywords:** epigenetic biological age, biological age clock, DNA methylation, epigenetic markers, lifestyle patterns

## Abstract

Background: Sustaining long-term lifestyle change remains a major challenge in preventive health. Epigenetic clocks offer a dynamic, modifiable measure of biological ageing that may enhance motivation when returned to individuals. Objectives: This study had two aims: (1) to evaluate whether personalised health reports integrating epigenetic age, polygenic cancer risk scores, and lifestyle metrics could motivate sustained behavioural change; and (2) to examine variability across epigenetic clock generations to inform the selection of a suitable model for participant feedback. Methods: A total of 178 adults were recruited via the Graham Fulford Charitable Trust community testing programme, and 91 completed a one-year follow-up survey assessing behavioural, psychological, and knowledge-related outcomes. DNA methylation data from 140 samples were used to compare 14 epigenetic clocks across four generations. Results: Most participants reported positive lifestyle changes, including feeling healthier (72.5%), increased physical activity (60.4%), and improved diet (47.3%). Gains were also observed in health knowledge (63.7%) and psychological well-being (31.9%). Epigenetic clock comparisons revealed substantial heterogeneity across models. Zhang2019-BLUP was selected as a stable and interpretable measure of biological age that can be readily communicated to participants, supporting empowerment and improved health literacy, rather than serving only as a risk prediction metric. Conclusions: Personalised biomarker feedback including epigenetic age combined with lifestyle and wearable data can support self-reported improvements in health-related behaviours. Community-based delivery through trusted local networks proved effective. The marked variation between epigenetic clocks highlights the importance of selecting models designed for clear communication when used in public-facing health interventions.

## 1. Introduction

The global burden of chronic diseases, including cardiovascular disease, type 2 diabetes, obesity, and cancer, continues to rise, reinforcing the urgent need for effective public health strategies that promote sustainable lifestyle changes. Despite widespread health promotion initiatives, maintaining long-term adherence to healthy behaviours remains a persistent challenge. Individuals often struggle to sustain improved dietary habits, regular physical activity, and cessation of harmful behaviours like tobacco use and excessive alcohol consumption, creating a significant adherence gap in lifestyle interventions [1,2,3]. This challenge necessitates novel approaches that can effectively promote lasting behavioural change.

Epigenetic clocks predict biological age using DNA methylation patterns, and researchers have categorised them into four generations with distinct designs and purposes [4]. Building on this, recent work in ageing research highlights biological ageing as a more meaningful health indicator than chronological age. Beyond their use in epigenetic clocks, DNA methylation (DNAm) patterns also capture broader aspects of physiological decline and can signal an individual’s susceptibility to age-related diseases [5,6].

First-generation epigenetic clocks were built from cross-sectional data to predict chronological age. A milestone in this generation was Horvath’s pan-tissue clock [7], followed by other widely used models including the Hannum clock [8], Lin clock [9], Vidal-Bralo clock [10], Skin & Blood clock [11], and the Zhang clock [12], which improved age-prediction precision using Elastic Net (EN) and Best Linear Unbiased Prediction (BLUP) models.

Second-generation clocks (PhenoAge, GrimAge, GrimAgeV2) incorporate clinical biomarkers alongside DNA methylation to capture biological age and improve prediction of time to mortality [13,14,15].

Third-generation clocks, such as DunedinPACE, use longitudinal data to estimate the pace of ageing rather than absolute age [16].

The newest fourth-generation “causal clocks” (CausAge, AdaptAge, DamAge) select CpG sites with evidence of causal links to ageing-related outcomes, aiming to enhance biological relevance [4,17].

DNA methylation-based biological age assessment has demonstrated promising reversibility in response to lifestyle and behavioural modifications. Two randomised controlled trials found measurable changes in biological age after targeted interventions, indicating the dynamic nature of these epigenetic markers [18,19]. Supporting this concept, a recent systematic review and meta-analysis by Chervova et al. demonstrated that epigenetic age acceleration, as measured across multiple DNA methylation-based clocks, is statistically associated with increased risks of all-cause mortality, cardiovascular disease, cancer, and diabetes [20]. Moreover, meta-analyses indicate that each 5-year increase in DNA methylation age is associated with an 8 to 15% increased risk of mortality [21]. These findings suggest that biological age could be a powerful feedback tool to encourage healthier habits, as the idea of “reversing” age can be highly motivating.

Unlike first-generation clocks like Horvath and Hannum’s, which were based mainly on chronological age, advanced second- and third-generation clocks such as GrimAge, PhenoAge, and Dunedin Pace of Aging are more sensitive indicators of overall mortality risk and age-related conditions [4]. These dynamic clocks help link lifestyle behaviours to the pace of ageing, offering promising potential for personalised health interventions.

While genomic data holds promise for personalised medicine, its application for motivating sustained lifestyle change faces its own challenges. The key distinction lies in the perceived actionability of the information. Genomic data often reflects inherent predispositions that may be perceived as static or unchangeable. Even when genetic risk information meets established criteria for clinical utility, it can have unintended psychological effects [22]. As observed in our iHelp study and other research [23,24], genetic risk disclosure may sometimes be interpreted deterministically, reducing perceived behavioural control, while low perceived genetic risk may lead to complacency in preventive behaviours.

In contrast, epigenetic data offers distinct advantages for behavioural interventions. As a dynamic and modifiable biomarker, epigenetic age responds directly to lifestyle choices and environmental factors. This characteristic addresses the perceived biomarker information by providing feedback on measures that individuals can influence through their actions. This direct link between behaviour and biomarkers promotes a sense of control and empowerment, helping to motivate and sustain healthy lifestyle changes [18,19].

Based on this foundation, the EU-funded iHelp project was designed to address the persistent challenge of motivating sustained lifestyle change for cancer prevention by providing insightful feedback on biomarkers and lifestyle factors, including diet, physical activity, and sleep duration [25]. As part of the iHelp project, we have published our findings on the relationships between lifestyle patterns and epigenetic biological age measures in men, highlighting the potential impact of specific lifestyle-related factors on biological ageing. These results can serve as a reference for applying healthy lifestyle improvements in future disease prevention studies [26].

The first objective of this study is to determine whether personalised health reports combining biological age estimates from validated epigenetic clocks, individual polygenic cancer risk profiles, and lifestyle-based health scores can motivate sustained lifestyle change and improve engagement in preventive health programmes.

The second objective is to examine the variability of different epigenetic clocks across generations and to assess their relative strengths and limitations, enabling a clear rationale for selecting the most appropriate clock for communicating biological ageing information to community participants.

This manuscript intentionally adopts a mixed-methods, interdisciplinary approach, combining (i) biological measurements, including DNA methylation-based epigenetic clocks, with (ii) participant-reported outcomes assessing engagement, understanding, and perceived behavioural change following a personalised health feedback intervention. The aim is not to test biological mechanisms or psychological theory in isolation but rather to evaluate the real-world feasibility and perceived impact of returning complex biomarker information within a community-based setting.

## 2. Results

For the first objective, we presented the results based on the 91 respondents (51.1% response rate) who completed the questionnaire.

**Figure 1:**
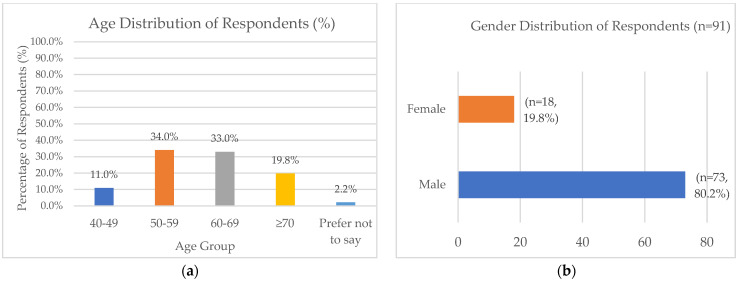
Demographic characteristics of study respondents. (**a**) Age distribution of respondents (n = 91). Most participants were aged between 50 and 69 years. (**b**) Gender distribution showing a higher proportion of male respondents (80.2%) compared to females (19.8%).

Figure 1a illustrates the age distribution of study respondents. The mean age of the respondents was 61.4 years old. The participant age distribution shows a representation from middle to older adulthood, with the majority in the 50–59 (34.1%, n = 31) and 60–69 (33.0%, n = 30) age groups. Respondents aged 70 and above contributed approximately 20% and around 10% of them were between 40 and 49 years old.

Figure 1b demonstrates the gender distribution of the study population. Males comprised the majority of respondents (n = 73), with only 18 participants identifying as female.

### 2.1. Feedback on Physical Aspect

The majority of the respondents stated that the iHelp project has positively influenced them on health-related behaviours (Table 1). More than 70% of the participants felt that iHelp project influenced them to become healthier, and around 60% reported being encouraged to take up more exercise. In terms of dietary habits, 47.3% agreed that the project encouraged them to have a better diet, whereas 39.6% agreed. Although both impact on smoking and alcohol consumption are less significant, only a minority (3.3% and 26.4%, respectively) reported behavioural changes after joining the project, with 42.9% of respondents agreed feeling healthier than before joining the project. Lastly, more than half agreed that the iHelp project did enough to keep them engaged.

### 2.2. Feedback on Psychological Aspect

The results of iHelp’s impact on the psychosocial aspect are shown in Table 2. A significant proportion of participants agreed that the iHelp project made a positive change in their happiness. Around 65% of the respondents reported that they became happier to some degree while more than 30% reported that they felt happier than before participating in the iHelp project.

### 2.3. Health Knowledge

The majority of participants (nearly two-thirds) felt that the iHelp project positively impacted their understanding of how to improve their health. A smaller proportion remained neutral, while less than 10% did not perceive a benefit in this area (Table 3).

Two open-ended questions were asked: What do you think the iHelp project did well? What do you think iHelp could have done better? Overall, participants reported a highly positive experience with the iHelp project. They particularly valued receiving personalised biomarker information that they could understand and act upon, with many finding the individualised feedback meaningful and motivating. The integration of Fitbit devices was also frequently mentioned as beneficial, helping participants track their daily activity and maintain motivation for lifestyle changes.

Suggestions for improvement centred on enhancing ongoing communication and support. Participants expressed strong interest in more regular contact through newsletters and updates that would help maintain their engagement with the epigenetic age concept and lifestyle modifications. Many requested clearer guidance on which specific lifestyle interventions would be most effective for improving their individual biological age.

### 2.4. Exploration of Epigenetic Clock Generations

For the second objective, we analysed DNA methylation data from 140 samples to explore variations in various epigenetic clocks in predicting biological age. The samples consisted of older adults with a mean age of 61.6 years (SD = 8.74); 90.7% were male and 9.3% were female (Table 4).

**Figure 2:**
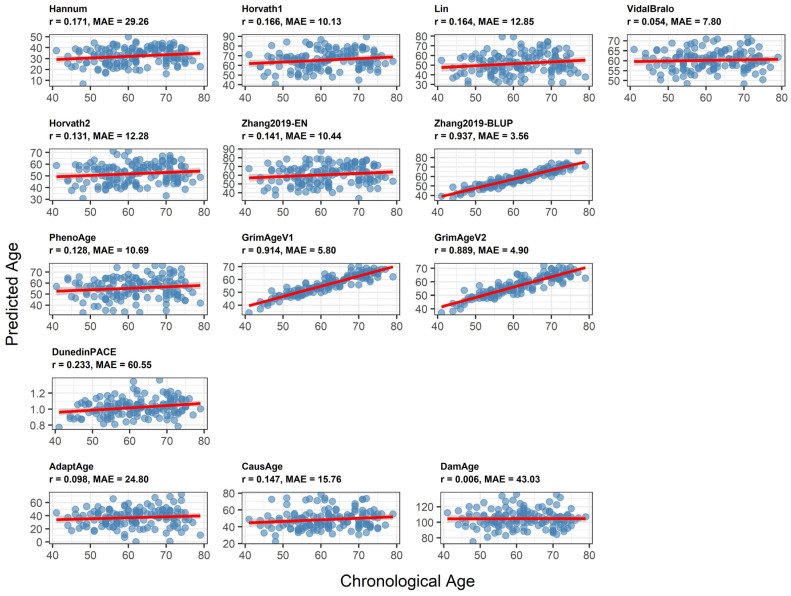
Scatter plots of chronological age to predicted age across 14 clocks. Each scatter plot reports Pearson correlation (r) and mean absolute error (MAE). The x-axis reports chronological age at the time of blood sample collection. The y-axis reports the age estimated by epigenetic clocks. If the points fall along the diagonal line y = x, the epigenetic clock accurately predicts the chronological age.

In predicting chronological age, the Zhang2019-BLUP (r = 0.937, MAE = 3.56), GrimAgeV1 (r = 0.914, MAE = 5.80) and GrimAgeV2 (r = 0.889, MAE = 4.90) clocks showed high correlations and low mean absolute errors (MAE). In contrast, the first-generation clocks displayed substantial heterogeneity in their outputs, with weak correlations and large mean absolute errors (MAE). Across all clock models, MAE varied markedly from approximately 10 to more than 40 years, illustrating wide differences in the biological age estimates they produce. Low correlations were also observed for the second- to fourth-generation clocks (PhenoAge, DunedinPACE, AdaptAge, CausAge, and DamAge), further emphasising the considerable variation between clocks in how they capture ageing-related signals. Scatter plots of chronological age to predicted age across 14 clocks are depicted in Figure 2.

**Figure 3:**
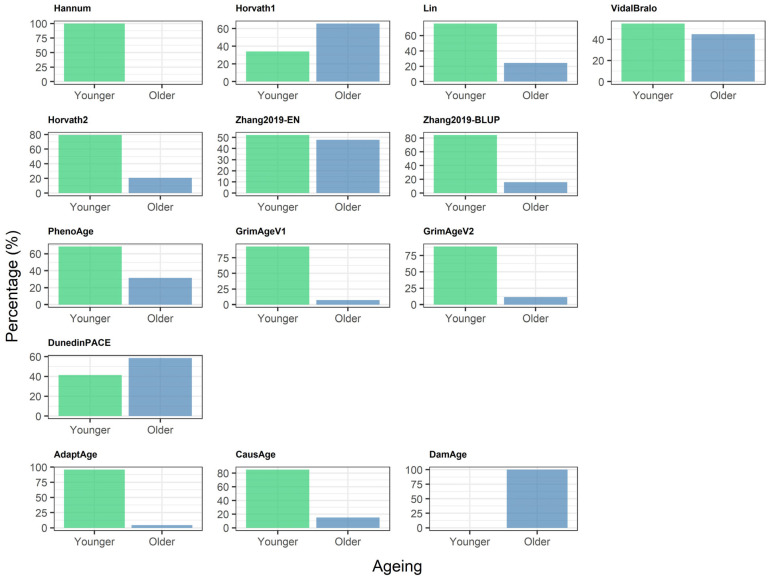
Distribution of epigenetic age acceleration versus deceleration across 14 clocks. The x-axis reports the categories of epigenetic age deviation, “Younger” (predicted age < chronological age) and “Older” (predicted age > chronological age). The y-axis reports the proportion of samples falling into each category.

The majority of clocks showed that participants’ epigenetic ages were younger than their chronological ages, indicating a general trend of age deceleration in this sample. However, three clocks exhibited oposite patterns: Horvath1, DunedinPACE, and DamAge showed a higher proportion of participants with accelerated than the decelerated ageing (Figure 3).

**Figure 4:**
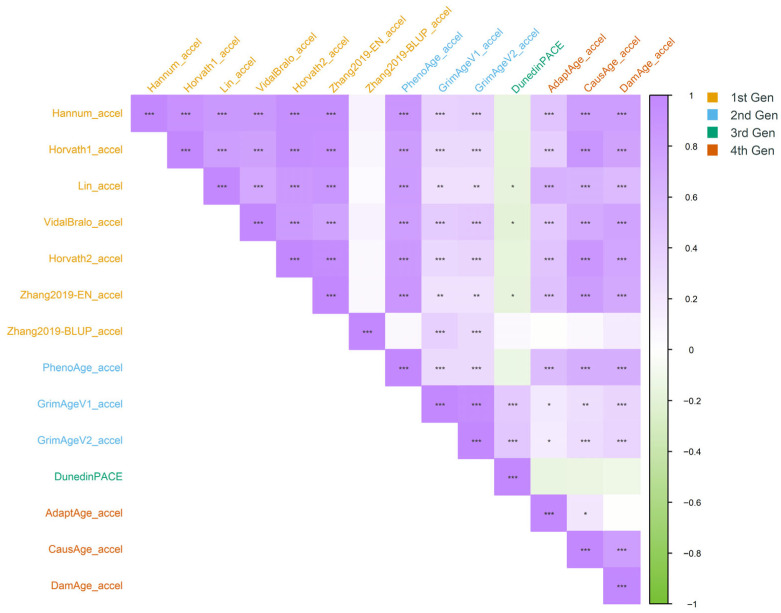
Correlation heatmap between age acceleration values (predicted age—chronological age) across 14 clocks. Significance levels are indicated as follows: *p* < 0.001 (***), *p* < 0.01 (**), and *p* < 0.05 (*).

The correlations of age acceleration values (predicted age—chronological age) across 14 epigenetic clocks are shown in Figure 4. Clocks from the same generation generally showed stronger correlations with each other than with clocks from different generations. Within the first generation, most clocks were highly inter-correlated, except for Zhang2019-BLUP, which showed weaker correlations likely due to methodological differences. Among the second-generation clocks, GrimAgeV1 and GrimAgeV2 were highly correlated because V2 is an updated version of V1, while PhenoAge showed weaker correlations with the GrimAge clocks because they capture different mortality-related mechanisms. The third-generation clock DunedinPACE exhibited weak negative correlations with most other clocks, consistent with its measurement of the rate of ageing rather than biological age state, but moderate positive correlations were observed with the GrimAge clocks. In the fourth generation, CausAge and DamAge were strongly correlated, while AdaptAge correlated weakly or not at all with them, as these models capture largely independent biological processes. The characteristics, design approaches, and clinical applications of each epigenetic clock are summarised in Table 5. Across generations, PhenoAge uniquely showed strong correlations with first-generation clocks (except Zhang2019-BLUP).

## 3. Discussion

The World Health Organization emphasised that healthy ageing is not merely the absence of disease but the process of developing and maintaining the functional ability that enables well-being in older age. With a globally ageing population, there is an increasing need for adaptable and personalised health interventions that support individuals through major life transitions [27]. With the development of epigenetic clocks, a new opportunity has emerged to provide individuals with biological ageing information as part of broader personalised feedback interventions that may support motivation for healthier lifestyle behaviours and promote healthy ageing.

The EU-funded iHelp project was designed to address the persistent challenge of motivating sustained lifestyle changes for cancer prevention by integrating lifestyle score with personalised health feedback [25]. It focused on promoting behavioural change among older adults by using tools such as wearable devices and biomarker-based reports, with epigenetic clocks and genetic risk scores. Our previously published study, “Exploring the Relationships between Lifestyle Patterns and Epigenetic Biological Age Measures in Men”, demonstrated that healthier lifestyle behaviours including higher physical activity, better diet quality, and adequate sleep were significantly associated with lower epigenetic biological age. These findings highlight the measurable impact of lifestyle on biological ageing processes [26].

It is important to emphasise that the iHelp intervention was inherently multicomponent, integrating epigenetic age estimates, polygenic cancer risk scores, lifestyle metrics, and wearable-derived activity feedback. As this study employed an observational follow-up design without a control group or factorial structure, the independent contribution of any single feedback component, including epigenetic age, cannot be isolated. The findings should therefore be interpreted as reflecting the combined effect of the personalised feedback package, rather than the causal impact of epigenetic age information alone.

The one-year follow-up results from the iHelp project suggest that personalised health feedback and wearable technology may support self-reported health behaviour change among midlife and older adults. Our findings reveal substantial improvements in health perception (72.5%), physical activity (60.4%), and health knowledge (63.7%), indicating how individualised biomarker information integrated with digital technology can support healthier ageing.

Providing participants with personalised biomarker feedback alongside lifestyle and physical activity data proved effective in promoting healthier behaviours, with 72.5% agreeing that the iHelp project influenced them to become healthier. This underscores the motivational power of personalised health feedback. Participants consistently cited the comprehensive report, including epigenetic clocks, genetic risk scores, and behavioural assessments, as a compelling driver for lifestyle change.

The observed improvements in general health perception and physical activity align with previous research on digital health interventions in older adults [28,29]. However, the modest improvements in dietary habits (47.3%) and minimal changes in smoking (3.3%) and alcohol consumption (26.4%) suggest that certain behaviours require more intensive intervention strategies. These findings are consistent with other behaviour change studies, which showed that long-established habits often need more intensive support than just providing information [30,31].

This “communicative power” of personalised biomarkers together with their lifestyle behaviour score effectively translated complex biological information into comprehensible, personal health metrics that participants could act upon across four key domains. These results demonstrate that a comprehensive feedback package is intuitively accessible to participants, successfully influencing their health perceptions.

The substantial increase in health knowledge (63.7%) and self-reported happiness (31.9%) demonstrates that the iHelp project serves dual functions, enhancing both participants’ understanding of their health and their psychological well-being. Participants gained not only factual knowledge but also a sense of empowerment over their health trajectory. This finding suggests the use of complex biomarker data as a means of enhancing health literacy.

Our results are consistent with previous studies demonstrating the effectiveness of personalised biomarker feedback and digital health supports [18,19,32,33]. However, this study uniquely demonstrates the comparative advantage of combined biomarkers and lifestyle feedback in motivating behaviour change. While genetic risk scores have shown utility in clinical settings [22], our findings suggest they also generate some impact on behavioural change in population-level approaches. The preference for biomarker feedback aligns with self-determination theory, which emphasises the importance of perceived competence and control in motivating behaviour change. Evidence suggests that epigenetic age provides a metric that responds to individual actions, thereby satisfying the psychological need for autonomy and competence [34].

Epigenetic clocks demonstrate heterogeneity both within and across generations, which reflects various approaches to clock design and underlying methodologies. Figure 2, Figure 3 and Figure 4 are ordered by clock generation to highlight conceptual differences in clock design rather than to imply a monotonic gradient of performance; indeed, correlation with chronological age is expected to vary by design intent rather than generation. The observed heterogeneity in correlations and MAE across epigenetic clocks reflects fundamental differences in clock design and intended purpose. Importantly, lower correlation with chronological age does not imply lower biological relevance, particularly for second-, third-, and fourth-generation clocks that are designed to capture mortality risk, physiological decline, or the pace of ageing rather than chronological age estimation. Only Zhang et al. (2019), trained on 13,661 samples, showed improved robustness [12]. In our sample, most clocks showed a higher proportion of individuals with slower ageing compared to those experiencing accelerated ageing, whereas Horvath1, DunedinPACE, and Damage demonstrated the opposite pattern. This may be due to the biological variability that different generations of clocks capture. Moreover, within each generation, each clock selects a distinct subset of CpG sites and assigns different coefficients to reflect the importance of each site within that model. DunedinPACE showed weak or inverse correlations with most age-predicting clocks, which is expected given that it measures the rate of physiological decline rather than biological age state. As a longitudinal pace-of-ageing metric, its discordance with cross-sectional age estimators reflects conceptual rather than methodological disagreement.

Zhang2019-BLUP was selected as a stable and interpretable measure of biological age that can be readily communicated to participants, supporting empowerment and improved health literacy, rather than serving only as a risk prediction metric. This choice was not intended to imply superior biological or clinical validity relative to newer clocks, which may be more appropriate for disease risk prediction or ageing mechanism research. Its relatively close alignment with chronological age provides feedback that is easy to interpret and unlikely to generate unnecessary anxiety. Importantly, our intention was not to use an epigenetic clock to predict participants’ health outcomes but rather to provide a neutral and accessible biological age measure that could initiate and support constructive, engaging conversations between participants and health consultants about lifestyle choices.

This approach, however, comes with limitations. As the Zhang2019-BLUP clock becomes more accurate in estimating chronological age, it loses predictive power for mortality and health outcomes. Therefore, studies aiming to assess biological health status or disease risk may be better served by newer epigenetic clocks optimised for predicting clinically relevant outcomes rather than chronological age.

These findings have several important implications for the design of health promotion interventions. Firstly, personalised biomarkers combined with lifestyle feedback effectively motivate preventive health behaviours. This suggests that future public health initiatives could incorporate modifiable biological markers when designing personalised feedback systems. Secondly, the integration of wearable technology with personalised biomarker feedback appears to create an effective behaviour change environment. The positive feedback regarding Fitbit devices indicates that real-time monitoring, when combined with personalised biomarker feedback, may enhance participant engagement. However, as wearable data were not analysed as objective outcome measures in this study, conclusions regarding sustained changes in physical activity rely on self-reported data and should be interpreted with appropriate caution. Thirdly, the educational component of the intervention proved crucial, suggesting that fully understanding what the results mean and how to act upon them kept participants motivated.

When providing epigenetic clock feedback in community settings, it is important to prioritise models that offer clear and interpretable results and minimise the risk of misinterpretation, particularly when the primary aim is to use the feedback as a conversation-engaging tool. Clocks optimised for predicting mortality or disease risk can generate unnecessary concern and are therefore less suitable for participant-facing reports. In contrast, clocks that align closely with chronological age provide more neutral and accessible feedback, supporting constructive, engagement-focused discussions between participants and health consultants. Ensuring that the selected clock is validated, transparent, and produces stable estimates further promotes trust and facilitates effective communication. Consequently, models such as Zhang2019-BLUP are more appropriate for engagement-oriented feedback than newer clocks designed primarily for risk prediction.

Furthermore, this study also highlights the potential value of engaging community-based charities, such as the Graham Fulford Charitable Trust (https://gfct.mypsatests.org.uk/ (accessed on 5 June 2025)), in the delivery of personalised health testing. Their established community presence and trusted relationships can enhance outreach, particularly among populations who may be less likely to engage with conventional healthcare services. By engaging with local networks, these organisations can facilitate recruitment, improve participant retention, and contribute to more equitable access to preventive health initiatives. This model may be particularly effective for future large-scale screening or health improvement programmes aiming to reach diverse and underserved communities.

Several limitations must be considered when interpreting these results. The predominant male representation (80.2%) substantially limits the generalisability of the findings, particularly for dietary behaviours and psychological outcomes, where sex-specific differences are well documented [35,36,37]. Conclusions regarding broader community applicability should therefore be interpreted cautiously. Future research should adjust recruitment strategies to better reflect the wider population.

The behavioural outcomes are presented as descriptive, self-reported follow-up measures rather than inferential tests of efficacy. Accordingly, this study was not designed or powered to test statistically significant behavioural change, and the absence of baseline measurements using the same instruments or a control group precludes formal hypothesis testing. Furthermore, age and gender may plausibly influence behavioural responses; the study sample particularly for female participants was small and highly unbalanced. Under these conditions, regression modelling or subgroup analyses would introduce unstable estimates and over-interpretation; therefore, we adopted a transparent descriptive approach and did not conduct age- or sex-stratified statistical analyses.

Moreover, the self-reported nature of behaviour change outcomes may introduce response bias, and objective measures of behaviour change would enhance the robustness of future studies.

A further key limitation of this study is the inability to disentangle the specific contributions of each biomarker type, epigenetic age, polygenic risk scores, and lifestyle metrics to the observed behavioural changes. The reported findings therefore should be interpreted as reflecting the overall personalised feedback intervention rather than the effect of epigenetic age information in isolation. This study was observational and not designed to disentangle the independent contribution of individual components, and causal attribution to epigenetic age feedback alone is therefore not justified. Instead, the observed associations likely arise from the combined influence of integrated risk communication and participant engagement with the broader feedback framework.

Future studies should consider experimental designs that enable a deeper understanding of which feedback elements are most effective, and under what conditions, to inform the optimisation of personalised prevention strategies.

The Hawthorne effect cannot be ruled out. The Hawthorne effect emphasises the complexity of human behaviour and the challenges of conducting research that accurately reflects real-world settings. Research participants may change their behaviour simply because they are aware of being observed. This can lead to results that are not truly representative of normal behaviour or clinical outcomes, thereby reducing the external validity of the study [38].

These findings have directly informed the design of the EU/UKRI-funded COMFORTage trial (https://comfortage.eu/ (accessed on 5 June 2025)), which will address several limitations of the current study [39]. The expanded biomarker panel will include multiple ageing-related measures, and the inclusion of cognitive and functional ageing outcomes will provide a more comprehensive assessment of biomarkers effectiveness.

The iHelp project demonstrates that personalised biomarker feedback combined with lifestyle information represents a promising approach for promoting healthy ageing behaviours. This study addresses a gap in the literature, as no previous research has examined the impact of feeding back comprehensive biomarkers alongside lifestyle factors.

## 4. Materials and Methods

The iHelp project was a university-led, ethically approved research study evaluating personalised cancer risk assessment with pancreatic cancer risk in both sexes, sex-specific assessment of breast cancer in females, prostate cancer in males and prevention in community-based adults, with a focus on integrating lifestyle, demographic, genetic, and epigenetic information to support health-promoting behaviour. Participants completed an online risk assessment generating a non-diagnostic estimate of future cancer risk. Participants with elevated absolute cancer risk compared with the general population were invited to provide blood samples for assessment of genetic risk and epigenetic age. These participants also received a written personalised report summarising their objectively measured physical activity (via Fitbit), self-reported diet (via Food Frequency Questionnaire), and genetic and epigenetic age metrics. Additional explanation of the results was offered through a follow-up telephone call upon participant request. The study was observational and multicomponent, designed to examine how integrated risk and biological ageing feedback may influence participant understanding and engagement to change their behaviour. The project was funded by the European Union’s Horizon 2020 research and innovation programme.

### 4.1. Participants Selection

**Figure 5:**
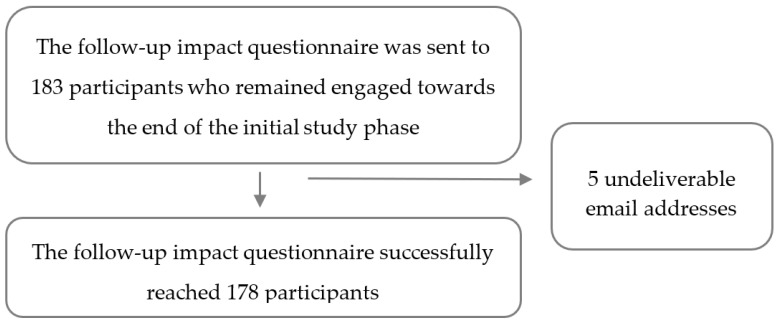
The flowchart of the recipient selection and email delivery process.

Participants were drawn from the iHelp project, which recruited individuals through a community-based approach led by the Graham Fulford Charitable Trust. Those who remained engaged throughout the study duration were included in this follow-up analysis. To address our first objective, a comprehensive impact assessment questionnaire was electronically distributed to 183 individuals who had participated in the study for one year. Of these, five emails were undeliverable due to invalid addresses, resulting in successful delivery to 178 participants. The participant selection process and email distribution methodology are detailed in Figure 5.

At enrolment, participants were informed that they were taking part in a university-led, ethically approved research study evaluating personalised cancer risk assessment and prevention, distinct from clinical screening or medical care. This study involved completion of an online cancer risk assessment tool (REFLECT) based on self-reported demographic, lifestyle, health, and family history data, with results presented as a non-diagnostic estimate of future cancer risk relative to the age-specific population average. The assessment was expected to take approximately 30 min and was described as probabilistic and potentially modifiable through lifestyle factors. Participants were informed that results would not be communicated to healthcare providers and would not lead to clinical intervention. Those identified as having above-average estimated risk were informed that they might be invited to optional additional study components, including biological measurements and a prevention programme, with participation entirely voluntary. All participants were informed of their right to withdraw at any time and of secure, pseudonymised data handling with anonymised dissemination of findings.

As part of the iHelp project, participants received personalised health reports that combined biological age estimates from epigenetic clocks, polygenic risk scores for prostate (males), breast (females), and pancreatic cancer, and lifestyle-based feedback. Physical activity data collected via Fitbit devices were also incorporated, providing a comprehensive and individualised overview of biological and behavioural risk factors.

To address our second objective, we selected a subset of 140 participants from the iHelp cohort for epigenetic clock exploration. These individuals were chosen based on the availability of high-quality blood samples collected during the initial biomarker assessment phase. DNA extracted from these samples was analysed using the Infinium Methylation EPIC v2.0 BeadChip (Illumina, San Diego, CA, USA), enabling the calculation of 14 epigenetic clocks spanning four generations. This subset was not restricted by behavioural survey participation; instead, it was selected to ensure adequate sample quality and representation for evaluating variability across clock models.

### 4.2. Data Collection and Measurements (First Objective)

To assess the long-term motivational impact, follow-up questionnaires were distributed to participants who remained engaged throughout the study period. This component of the study, corresponding to the first objective, focuses on the 178 British adults aged 40–79 who remained engaged through the one-year follow-up period. Behavioural, psychological, and knowledge-related outcomes were analysed based on the 91 participants (51.1%) who completed the online survey. The survey instrument was structured to capture self-reported data across three crucial domains: behavioural change (e.g., improvements in physical activity, diet, smoking, alcohol use), psychological well-being (e.g., perceived happiness and overall mental state), and the perceived impact of the feedback on their health understanding and motivation. Questions specifically designed to evaluate lifestyle behaviour changes in physical health, psychological state, and health knowledge resulted from iHelp project participation.

The questionnaire comprised nine mandatory multiple-choice questions and two optional open-ended questions on project strengths and areas for improvement. Seven questions were asked with regard to the physical domain while one question targeted psychological aspect and health knowledge, respectively. Responses to multiple-choice questions were measured using a 5-point Likert scale. Participant gender and age were obtained from the study database using assigned record IDs. This demographic information helps us contextualise the results and identify potential differences in perceptions based on participant characteristics.

The follow-up questionnaire was developed by the iHelp study team to evaluate participant perceptions of engagement, health knowledge, and self-reported behavioural change following the personalised feedback intervention. The instrument was adapted from an internal questionnaire previously used within the iHelp project and refined for one-year follow-up. The questionnaire was administered electronically using the REDCap (Research Electronic Data Capture) platform hosted at the University of Manchester (https://redcap.manchester.ac.uk/, accessed on 5 June 2025). REDCap is a secure, web-based tool specifically designed for research data collection, offering an intuitive interface, audit trails, automated data exports, and integration capabilities. A unique questionnaire link, embedding each participant’s assigned ID, was generated via REDCap, and this enabled efficient response tracking and automated reminders to optimise completion rates from the 178 engaged participants.

### 4.3. Epigenetic Clock Analysis (Second Objective)

Genome-wide DNA methylation profiling was performed using the Infinium Methylation EPIC v2.0 BeadChip (Illumina Inc., San Diego, CA, USA), a microarray-based bead chip assay interrogating approximately 935,000 CpG sites. DNA extraction, bisulfite conversion, array hybridisation, and scanning were conducted according to the manufacturer’s standard protocols. The laboratory processing was performed by an accredited external service provider.

Quality control and data preprocessing were conducted by the authors using the Meffil R package, including probe- and sample-level filtering based on detection *p*-values, bead counts, signal intensity thresholds, and control probe performance. Batch effects were assessed and adjusted using functional normalisation prior to downstream epigenetic clock estimation.

To address the second objective, DNA methylation-based epigenetic age estimates were derived from blood samples using multiple established epigenetic clock models, which were calculated analytically for comparison and validation purposes. These included several widely used clocks to assess biological ageing and age acceleration across participants. However, only a single epigenetic clock, the Zhang2019-BLUP model, was selected for return of individual-level feedback to participants. The multi-clock analyses were conducted solely for internal evaluation, to inform clock selection and aid interpretation of biological ageing measures, and were not used for participant-facing reporting or clinical decision-making.

We evaluated variability across epigenetic clock generations using DNA methylation data from 140 selected iHelp participants. DNA was extracted from whole blood and processed using the Infinium Methylation EPIC v2.0 BeadChip (935,000 CpGs) [29], generating genome-wide methylation profiles suitable for multi-clock comparison. Pre-processing, quality control, data harmonisation, and normalisation were performed using the Meffil R package, which supports Infinium 450 K, EPIC v1, and EPIC v2 BeadChip [16,40]. During quality control, probes with detection *p*-values > 0.01 or bead counts <3 were excluded from downstream analysis. Probes failing signal intensity (±3 SD) or control probe (±5 SD) thresholds were similarly removed. Low-quality samples (outliers) were retained because the aim of this objective was exploratory—to capture the full range of epigenetic age variation across clock models rather than to refine sample representativeness. Normalised beta values were used to calculate 14 epigenetic clocks spanning four generations, enabling comparisons of predicted biological age, age acceleration, and inter-clock correlations.

Functional normalisation (FN) was applied to adjust for technical and batch-related variation by modelling the top principal components (PCs) derived from control probes and Sentrix row and column information. Batch effects were assessed through regression analyses of control-probe PCs against batch variables, with significant associations detected (*p* < 1 × 10^−50^). The resulting quantile residuals were retained as normalised quantiles for each sample, generating the final normalised beta values [40].

Using these beta values, we examined the association between chronological age and predicted epigenetic age, the distribution of epigenetic age acceleration and deceleration, and the inter-clock correlations across 14 epigenetic clocks representing four generations. These included first-generation clocks (Hannum, 2013 [8]; Horvath1, 2013 [7]; Lin, 2016 [9]; Vidal-Bralo, 2016 [10]; Horvath2, 2018 [11]; Zhang-EN, 2019 [12]; Zhang-BLUP, 2019 [12]), second-generation clocks (PhenoAge, 2018 [13]; GrimAgeV1, 2019 [15]; GrimAgeV2, 2022 [14]), a third-generation pace-of-ageing measure (DunedinPACE, 2022 [16]), and fourth-generation clocks (AdaptAge, 2024 [17]; CausAge, 2024 [17]; DamAge, 2024 [17]).

### 4.4. Data Analysis

**Figure 6:**
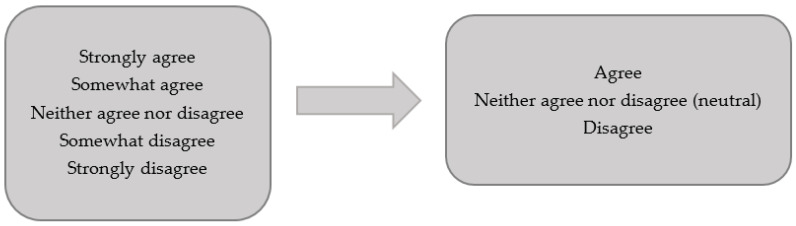
Conversion of 5-point Likert scale to three groups.

For the survey, responses on REDcap were exported to Microsoft Excel (.xls) files. Demographic variables were summarised descriptively to provide contextual information; given the small and highly unbalanced number of female participants; sex-stratified analyses or distributional visualisations (e.g., violin plots) were not performed, as these would risk misleading interpretation. To simplify analysis while maintaining data integrity, the original 5-point Likert scale [41] responses were grouped into three categories. “Strongly agree” and “agree” were combined into an “agree” category, while “strongly disagree” and “disagree” were combined into a “disagree” category. “Neither agree nor disagree” remained as its own category. This approach made data analysis more straightforward without losing important information about participants’ perceptions (see Figure 6).

However, a “N/A” option was included for two questions about smoking and alcohol consumption to account for participants who are not smokers or heavy drinkers.

Regarding the open-ended questions, content analysis was employed to systematically categorise responses by identifying specific words, phrases, and thematic concepts. Responses were exported into Excel files, and each answer was reviewed, categorised and calculated for frequency analysis.

The chronological age was calculated using Hannum [8], Horvath1 [7], Lin, VidalBralo [10], Horvath2 [11], Zhang2019-EN [12], Zhang2019-BLUP [12], PhenoAge [13], GrimAge [15], DunedinPACE [16], AdaptAge [17], CausAge [17], DamAge [17] implemented via the methylCIPHER [15,42] R package and DunedinPACE [16] implemented via the dnaMethyAge R package [43]. To evaluate the compatibility between the selected epigenetic clocks and the dataset, CpG probe overlap was assessed (percentage of match with the input beta values). The average present CpG rate in the samples across the clocks is shown in Table 6. We also calculated the percentage of individuals who showed accelerated or decelerated ageing across the generation of clocks. The correlations of age acceleration values (predicted age—chronological age) across 14 epigenetic clocks were calculated. All epigenetic data analyses were carried out in RStudio version 4.5.1 [44]. 

## 5. Conclusions

This one-year follow-up from the iHelp project indicates that a multicomponent personalised feedback intervention integrating epigenetic age estimates, genetic risk information, lifestyle assessment, and wearable-derived activity tracking was associated with self-reported improvements in health perception, physical activity, and health knowledge among participants who remained engaged. These findings support the feasibility of returning complex biomarker information within a structured community-based setting and suggest that participants perceive such feedback as meaningful and motivating.

Importantly, the observational and multicomponent design does not allow attribution of behavioural changes to any single feedback element, including epigenetic age information. The results therefore reflect participant-reported responses to the integrated personalised feedback package rather than evidence of causal impact.

Substantial heterogeneity across epigenetic clocks was observed, reinforcing that clock selection is critical when biological ageing information is communicated to non-clinical populations. Clocks optimised for alignment with chronological age, such as Zhang2019-BLUP, were more suitable for participant-facing communication due to their interpretability and stability. In contrast, clocks designed to capture mortality risk or pace of ageing may be more appropriate for research or clinical risk stratification rather than engagement-focused feedback. The findings further suggest that biomarker feedback may be most acceptable and engaging when accompanied by explanatory support and wearable-derived contextual data. The involvement of a trusted community organisation facilitated recruitment and sustained engagement, highlighting the potential value of community partnerships in preventive health research.

Taken together, this study provides evidence of feasibility and perceived behavioural impact of integrated biomarker feedback in a real-world setting. Future controlled and factorial studies will be required to determine the independent contribution of epigenetic age feedback and to evaluate objective behavioural outcomes and longer-term health effects.

## Figures and Tables

**Table 1:** Distribution of agreement in physical impact assessment.

Aspect	Impact Assessment Questions	Scale			
		Disagree	Neutral	Agree	N/A
**Physical** **(91)**	Do you feel the iHelp project influenced you to become healthier?	4 (4.4%)	21 (23.1%)	66 (72.5%)	0 (0%)
	Do you feel the iHelp project has encouraged you to take up more exercise?	11 (12.1%)	25 (27.5%)	55 (60.4%)	0 (0%)
	Do you think the iHelp project encouraged you to have a better diet?	12 (13.2%)	36 (39.6%)	43 (47.3%)	0 (0%)
	Do you feel the iHelp encouraged you to quit or reduce smoking?	6 (6.6%)	4 (4.4%)	3 (3.3%)	78 (85.7%)
	Do you feel the iHelp project encouraged you to reduce or stop alcohol consumption?	17 (18.7%)	40 (44.0%)	24 (26.4%)	10 (11.0%)
	Do you feel healthier now than before joining the iHelp project?	7 (7.7%)	45 (49.5%)	39 (42.9%)	0 (0%)
	Do you feel the iHelp project did enough to keep you engaged in the project?	13 (14.3%)	31 (34.1%)	47 (51.6%)	0 (0%)

**Table 2:** Agreement levels in psychological impact assessment.

Aspect	Impact Assessment Questions	Scale		
		Disagree	Neutral	Agree
**Psychological**	Do you feel happier now than before joining the iHelp project?	6 (6.6%)	56 (65.1%)	29 (31.9%)

**Table 3:** Participant agreement on health knowledge acquisition.

Aspect	Impact Assessment Questions	Scale		
		Disagree	Neutral	Agree
**Health** **Knowledge**	Do you feel the iHelp project helped you gain knowledge of how to improve your health?	9 (9.9%)	24 (26.4%)	58 (63.7%)

**Table 4:** Description of sample characteristics.

	Overall (N = 140)
Age	
Mean (SD)	61.6 (8.74)
Median [Min, Max]	62.0 [41.0, 79.0]
Gender	
Female	13 (9.3%)
Male	127 (90.7%)

**Table 5:** Comparison of epigenetic clock characteristics and functions across generations.

Generation	Clock Name	Predicted Outcome	Noof CpGs	Tissues Derived	Sample	Array Type Trained	Functions
**Generation 1**	Hannum (2013) [8]	Chronological Age	71	Blood	482	450 K	Reflects telomere length, metabolic and DNA repair pathway
	Horvath1 (2013) [7]		353	Multi-tissue	3931	450 K and 27 K	Associated with cancer
	Lin (2016) [9]		99	Blood	656	27 K	Associated with mortality risk
	VidalBralo (2016) [10]		8	Blood	390	27 K	An accessible epigenetic clock requires only 8 CpGs
	Horvath2 (2018) [11]		391	Human fibroblasts and endothelial cells divisions, skin, blood, and saliva	896	450 K and EPICv1	Predicts time to death;
							associated with immune system (naïve CD8+ T cells, naïve CD4 + T cells)
	Zhang2019-EN (2019) [12]		514	Blood and saliva	13,661	450 K	Increased prediction of chronological age
	Zhang2019-BLUP (2019) [12]		319,607	Blood and saliva	13,661	450 K	Increased prediction of chronological age
**Generation 2**	PhenoAge (2018) [13]	Mortality proxy built from clinical biomarkers	513	Blood	9926	450 K	Reflects all-cause mortality, morbidity and frailty, and immunosenescence (naïve CD8+ T cells, naïve CD4 + T cells, CD4+ helper T cells and B cells)
	GrimAge1 (2019) [15]	Mortality	1030	Blood	1731	450 K	Predicts all-cause mortality; associated with heart disease, immune system functioning (naïve CD8+ T cells, naïve CD4 + T cells, B cells), and leucocyte telomere length
	GrimAge2 (2022) [14]		1030	Blood	1833	450 K	Predicts all-cause mortality; associated with heart disease, time-to-any cancer, type 2 diabetes, hypertension, disease free status, physical functioning level
**Generation 3**	DunedinPACE (2022) [16]	Pace of Ageing	173	Blood	1037	450 K and EPICv1	Measures pace of ageing; Associated with morbidity, mortality, CVD, and disability
**Generation 4**	AdaptAge (2024) [17]	8 ageing-related phenotypes	1000	Blood	2644	450 K	Reflects protective change during ageing
	CausAge (2024) [17]		586	Blood	2644	450 K	Reflects accumulation of overall casual age changes link to mortality risks; detects hypertensive heart disease
	DamAge (2024) [17]		1090	Blood	2644	450 K	Reflects age-damaging biology

**Table 6:** Present CpG rate for each clock.

Clock	Total Probes	Present Probes	Present Probes %
Hannum	71	64	90%
Horvath1	353	340	96%
Lin	99	96	97%
VidalBralo	8	8	100%
Horvath2	391	374	96%
Zhang2019-EN	514	493	96%
Zhang2019-BLUP	319,607	287,256	90%
PhenoAge	513	495	96%
GrimAge	1030	845	82%
DunedinPACE	173	144	83%
AdaptAge	999	938	94%
CausAge	581	496	85%
DamAge	1089	1016	93%

## Data Availability

The data for this study is not publicly available due to privacy and ethical restrictions. Access to the data is limited to researchers who have received ethical review approval and consent from all the study subjects.
